# Effects of resilience training for mothers on maternal resilience and children’s pain in pediatric burn units in a randomized controlled trial

**DOI:** 10.1038/s41598-025-02040-9

**Published:** 2025-05-17

**Authors:** Fahimeh Alsadat Hosseini, Maryam Shaygan, Maryam Shayegan

**Affiliations:** 1 Community Based Psychiatric Care Research Center, School of Nursing and Midwifery, Shiraz University of Medical Sciences, Shiraz, Iran; 2 Department of Nursing, School of Nursing and Midwifery, Kazerun, Islamic Azad University, Kazerun, Iran

**Keywords:** Maternal behavior, Child, Burn injuries, Resilience, Pain management, Human behaviour, Pain

## Abstract

Mothers of children with burn injuries often experience psychological distress, affecting their well-being and children’s pain. This study evaluates the impact of resilience training on maternal resilience and child pain. This randomized clinical trial was conducted at Amir Al-Momenin Burn Hospital, Shiraz, Iran, with 50 mothers in 2021–2022. Participants were assigned to an intervention group (six-day resilience training) or a control group (standard care). Outcomes were measured at multiple time points using the Connor-Davidson Resilience Scale and the Visual Analog Scale. Data were analyzed using SPSS v.22. The analysis revealed significant time effects on child pain intensity (B = − 0.84, *p* < 0.001) and maternal resilience (B = 3.99, *p* < 0.001). Significant group effects revealed greater improvements in the intervention group for child pain intensity (B = 2.85, *p* < 0.001) and maternal resilience (B = − 3.05, *p* < 0.001). The intervention group showed significant improvement in maternal resilience over time compared to the control group (B= − 2.06, *p* = 0.001), with no significant difference in child pain intensity over time compared to the control group (B = − 0.05, *p* = 0.69). Resilience training enhances maternal resilience and children’s pain over time. However, its impact on child pain intensity is limited compared to standard care. Therefore, integrating resilience training for mothers into pediatric burn care is recommended.

## Introduction

Burn injuries represent a significant cause of morbidity and mortality in children^1^. It is estimated that 90% of these cases occur in low- and middle-income countries^1^. In Iran, the prevalence of burns among children is particularly high compared to other age groups, with approximately 3 million cases reported annually^2^.

Although burn injuries are common among children and their associated pain is severe, the management of burn pain remains insufficiently addressed^3^. Uncontrolled pain can have adverse effects on patients both in the short term and over the long term. It can induce anticipatory anxiety regarding future medical procedures, and heightened levels of pain and anxiety may impede wound re-epithelialization. This delay in healing can result in prolonged consequences affecting growth and mobility. Severe pain resulting from pediatric burns not only has significant psychological effects on the children but also exerts detrimental psychological impacts on their families^4^. Parents of children with burn injuries frequently experience feelings of guilt, blame, and shame^5^. They endure greater psychological distress compared to parents of children with other injuries or medical conditions^6^. While both mothers and fathers are profoundly impacted by their child’s burn injury^7^, mothers typically experience greater psychological distress compared to fathers^8,9^.

A substantial body of research indicates a high prevalence of psychological distress among mothers of burn-injured children. Studies consistently report elevated rates of post-traumatic stress disorder (PTSD), anxiety, and depression in these mothers^10–13^. Maternal psychological stress following pediatric burn injuries is associated with a range of adverse child outcomes. Children whose mothers experience significant stress may exhibit more emotional regulation difficulties, struggling to manage their emotions effectively^12–14^. In addition, maternal stress, in particular, has been identified as a critical factor that can exacerbate a child’s pain experience^15^. In line with this, studies show the positive effect of mothers’ psychological well-being on their children’s chronic pain^16,17^. In the field of pediatric burn injury, only one study by Brown et al. (2019) has shown that increased psychological support for parents may reduce procedural pain-related distress in young burn-injured children^18^. In this regard, providing the strategies to enhance maternal resilience seems crucial to alleviate the psychological burden on mothers and improve the mental and physical well-being of their children.

Resilience is characterized by adaptive and dynamic changes in cognitive, emotional, and behavioral domains that contribute to enhanced mental health^19^. Parents of children who survive burn injuries often undergo substantial psychological distress and exhibit low resilience^20^. Promoting resilience in mothers can buffer the negative effects of stress, leading to improved coping mechanisms and better outcomes for both the mother and the child^21^. Therefore, resilience training may be a beneficial strategy to enhance the resilience of mothers of children with burn injuries and reduce their children’s pain.

Resilience training is a structured psychological intervention designed to enhance adaptive coping and emotional regulation, enabling individuals to manage stress more effectively^22–24^. While there is a paucity of research specifically on resilience training for mothers of burn-injured children, the broader literature on resilience and stress management provides valuable insights^21^. In the context of burn injuries, it was shown that psychotherapy, including cognitive behavioral therapy (CBT) and other evidence-based approaches, can help mothers develop coping skills and manage their psychological distress^13,25^. In other contexts, research has shown that such training can significantly reduce psychological distress in mothers of children with chronic conditions, improving their emotional well-being and caregiving capacity^26–29^. However, no experimental studies have been found that assess the impact of resilience training for mothers on the pain experience of children with burn injuries. In this line, Brown et al. (2019) proposed a conceptual model within the pediatric burn unit framework, suggesting that variations in parental procedural behavior mediate the relationship between parental psychological distress and child procedural distress^30^. This model implies that enhancing acute psychological support for parents of burn-injured children can alleviate procedural pain-related distress in these young patients^30^. Given the well-established link between maternal stress and child health outcomes, reducing maternal distress may contribute to better pain management in children by fostering a more supportive and responsive caregiving environment^26^. However, there is a need for studies specifically assessing the impact of resilience training on both maternal resilience and the pain experience of children with burn injuries.

Given the high risk of psychological distress among mothers of children with burn injuries^31,32^ and its potential impact on their children’s pain^33,34^, it is crucial to assess effective psychological interventions. While existing studies underscore the benefits of parental resilience training^35^ and its potential to improve child pain outcomes^16,17,36,37^, most focus on chronic pain conditions other than burns. Therefore, this study aims to evaluate the impact of resilience training for mothers of hospitalized children with burn injuries on the resilience of mothers and the pain experienced by their burn-injured children, guided by the conceptual model established by Brown et al. (2019). We hypothesize that resilience training for these mothers will lead to significant improvements in maternal resilience and reductions in child pain, as assessed during and after the intervention and at follow-up points.

## Method

**Fig. 1:**
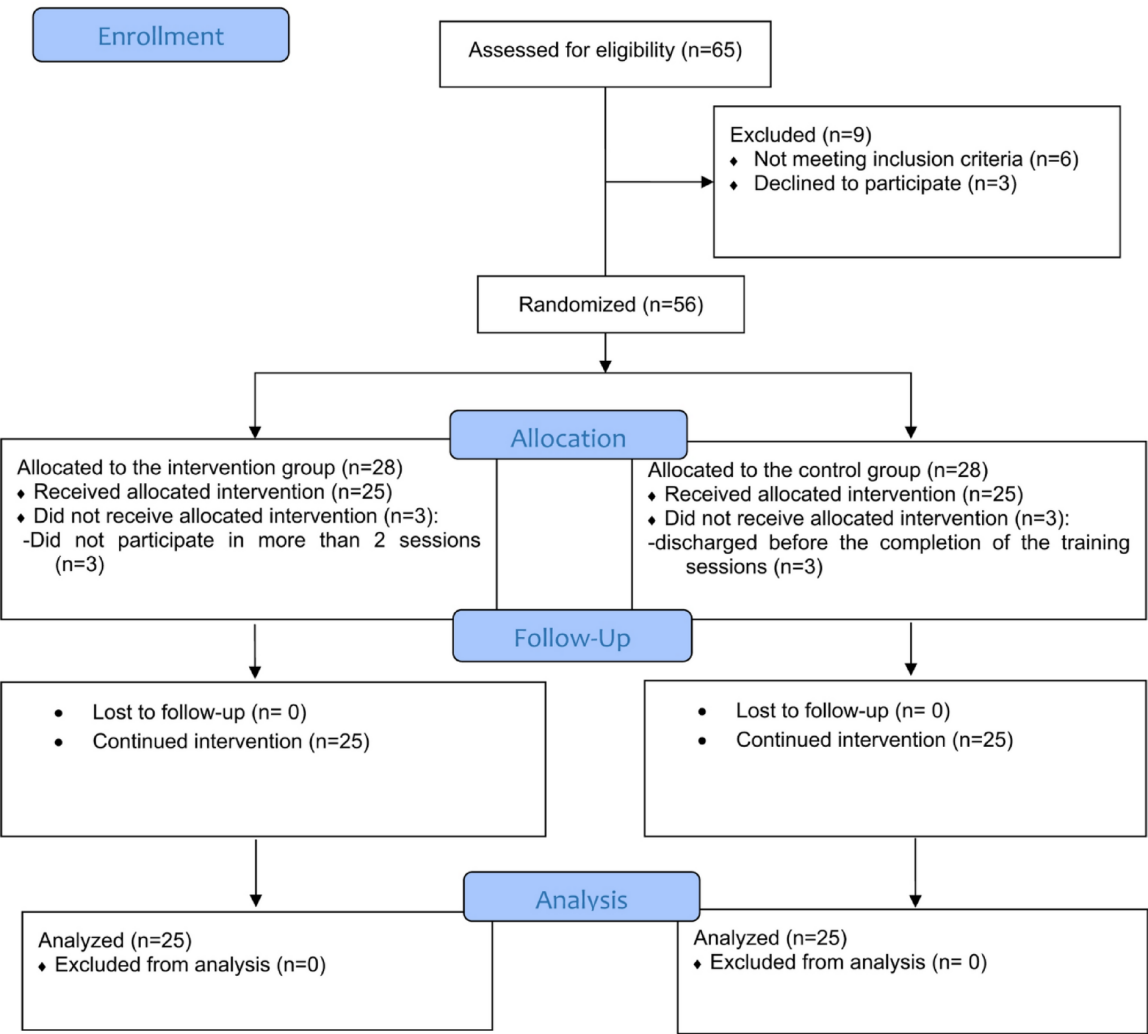
CONSORT flow diagram.

This study was conducted in accordance with the CONSORT guidelines^38,39^ [see the CONSORT diagram in Fig. 1].

### Study design

This study is designed as a parallel-group, double-blind, randomized clinical trial with comparator controls.

### Participants and settings

The study was conducted between November 2021 and April 2022 at the pediatric ward of Amir Al-Momenin Burn Hospital, affiliated with Shiraz University of Medical Sciences, in Shiraz, Iran. The participants consisted of 50 mothers of children aged 6 to 12 years who were hospitalized with burn injuries (degree 2 or greater) and their respective children. Inclusion criteria required that the children have a burn injury diagnosed by the treating physician, maternal literacy, and the willingness of both mothers and children to participate in the study. Exclusion criteria included chronic physical or mental illnesses in the mother or child, maternal absence from more than two resilience training sessions, pre-existing child pain before the burn injury, and severe fractures or non-burn-related wounds in the child.

Mothers of hospitalized children who met the inclusion criteria were approached by the principal researcher, and written informed consent was obtained from those willing to participate. After eligibility was confirmed, participants were randomly assigned to either the experimental group (*n* = 25) or the control group (*n* = 25) using a 1:1 random sampling method. The experimental group received the resilience training intervention, while the control group did not. Both groups completed a resilience questionnaire at three time points: before the first session, before the fourth session, and immediately after the final session. Additionally, participants completed the questionnaire daily until the fifteenth day of hospitalization, totaling 12 assessments at 5 p.m.

### Sample size calculation

Sample size was estimated using G*Power 3.1 software. Based on an α level of 0.05, a power of 0.90, and an effect size of 0.40 ^40^, the required sample size was 47. To compensate for a 20% anticipated dropout rate, 56 participants were recruited, and 50 completed the study.

### Randomization and blinding

To minimize bias and enhance the reliability of the findings, randomization and blinding were rigorously applied in this study. Children with burn injuries and their mothers were randomly assigned to either the intervention group, which received resilience training for the mothers, or the control group, which did not receive any resilience training. Randomization was performed using the randomizer.org tool, with participants randomly allocated to either the intervention or control group. This method of simple randomization was conducted by an independent researcher who was not involved in participant recruitment, intervention delivery, or outcome assessment. Each child and their corresponding mother were assigned a unique identification number. These numbers were entered into the randomization software, which then randomly allocated the participants into either the intervention or control group using these identifiers. The use of random allocation was crucial to eliminate selection bias and ensure that each participant had an equal chance of being assigned to either the intervention or control group, thereby enhancing the internal validity of the study.

To prevent data contamination, data collection from the control group was completed before data collection from the intervention group. This sequencing ensured that the control group’s data remained unaffected by the subsequent intervention and maintained the integrity of the data across both groups.

Blinding was rigorously implemented in this study to reduce the potential for bias. Participants were blinded to their group assignments, meaning they were unaware of whether they were part of the intervention or control group. This was critical to prevent any influence on their behavior, expectations, or responses that could arise from knowing their assigned group. Moreover, the intervention and control groups were designed to be indistinguishable to the participants, which further minimized the likelihood of bias related to their perception of the intervention. In addition, the outcome assessors and data analysts were also blinded to the group allocations. These individuals were responsible for collecting and interpreting the outcome data, and their lack of knowledge regarding the participants’ group assignments ensured that their assessments and analyses were not influenced by any preconceived expectations. By ensuring that both the participants and the evaluators remained blinded throughout the study, we were able to mitigate the risk of performance bias and detection bias, both of which are essential for maintaining the internal validity of the study. These measures were taken to minimize bias and ensure the reliability and validity of the study results^41^.

### Resilience intervention condition

After completing the data collection for the control group, mothers in the intervention group participated in a six-session resilience training program. The sessions focused on stress management (2 sessions), identifying and addressing cognitive distortions (2 sessions), and applying positive psychology techniques (2 sessions). These sessions were conducted in groups of 3–6 participants at the Amir Al-Momenin Burn Hospital’s conference hall, with each session lasting 60–90 min. The sessions were held daily over a six-day period. The program was led by a psychiatric nurse (the third author), who holds specialized training in mental health nursing and has received additional training in resilience-building techniques and therapeutic interventions for individuals facing psychological distress.

The first session served as a needs assessment to tailor the program to the participants’ specific requirements. Various instructional methods were used, including visual aids (charts, films, PowerPoint slides), interactive group discussions, and practical exercises. Each session began with a lecture by the nurse, followed by group discussions and exercises. Participants were encouraged to select the techniques most beneficial for them and integrate them into their daily routines, rather than attempting to practice all techniques at once. Feedback on the strategies’ effectiveness was solicited throughout the program. Table 1 outlines the objectives and content covered in each of the six training sessions.

**Table 1:** Goals and content of educational program^42–46^.

Sessions	Objectives	Content
1	Stress management: part 1	• Introduction to the program & participant familiarization—Overview of objectives, structure, and expectations; establishing rapport between facilitators and participants. • Resilience in mental health—Definition, significance in coping with adversity, and its role in enhancing emotional regulation and adaptive coping strategies. • Completion of informed consent & baseline assessment—Obtaining informed consent, collecting demographic data, and administering pre-intervention questionnaires. • Understanding stress & its effects—Definition, sources of stress, physiological and psychological impact, and its influence on maternal well-being and child pain perception. • Stress appraisal & coping mechanisms—Differentiating between adaptive and maladaptive coping strategies, exploring cognitive and behavioral approaches to stress management. • Q&A and group discussion—Identifying personal stressors, sharing experiences, and discussing practical coping techniques in a supportive environment. • Group training activities—Interactive exercises to enhance self-awareness of stress responses and introduction to basic relaxation techniques (deep breathing, progressive muscle relaxation).
2	Stress management: part 2	• Review of the previous session—Recap of key concepts from Session 1, with a focus on participants’ reflections and experiences in applying initial stress management techniques. • Advanced coping strategies for stress management—Introduction to evidence-based techniques such as deep breathing, guided meditation, visualization, and progressive muscle relaxation, with demonstrations and participant practice. • Cognitive-emotional interaction in stress response—Exploring the relationship between negative thoughts, emotions, and stress, identifying maladaptive thought patterns, and discussing their impact on mental and physical health. • Cognitive restructuring techniques—Training participants in recognizing, challenging, and modifying negative thoughts related to illness and caregiving stress, fostering more adaptive cognitive patterns. • Mindfulness-based stress reduction (MBSR)—Introduction to mindfulness principles, practical exercises for cultivating present-moment awareness, and strategies for incorporating mindfulness into daily life. • Q&A and group discussion—Encouraging participants to share their experiences, ask questions, and refine their individual coping strategies. • Group training activities—Interactive exercises designed to reinforce the use of relaxation techniques, mindfulness practices, and cognitive restructuring through practical, real-life scenarios.
3	Cognitive errors and its management: part 1	• Review of the previous session—Summary of key concepts from Session 2, with a focus on stress management techniques and their relation to cognitive errors. • Definition and causes of cognitive errors—Explanation of common cognitive errors (e.g., overgeneralization, catastrophizing) and factors that contribute to their development, particularly in the context of illness and caregiving stress. • Effects of cognitive errors on health—Discussion on how cognitive errors worsen stress, anxiety, and depression, leading to negative health outcomes and impairing coping abilities. • Identifying and addressing cognitive biases—Techniques for recognizing and challenging cognitive biases (e.g., thought-stopping, reframing) to promote more balanced thinking, especially regarding illness. • Adverse outcomes from illness-related cognitive errors—Exploration of the negative effects of illness-related cognitive errors on physical and psychological well-being. • Q&A and group discussion—Interactive session for participants to share concerns and experiences, addressing challenges in managing cognitive errors. • Group training activities—Exercises and role-play activities to help participants practice cognitive restructuring techniques and apply them to real-life situations.
4	Cognitive errors and their management: part 2	• Review of the previous session—Recap of key concepts from Session 3, focusing on cognitive errors and their impact on health. • Overview of relaxation techniques—Introduction to and demonstration of evidence-based relaxation methods, including progressive muscle relaxation, guided imagery, and diaphragmatic breathing, aimed at reducing stress and enhancing emotional regulation. • Q&A and group discussion—Opportunity for participants to discuss challenges, share experiences, and ask questions about the use of relaxation techniques in managing cognitive errors. • Group training activities—Practical exercises allowing participants to practice relaxation techniques in real-time, emphasizing their application in reducing cognitive distortions and improving emotional well-being.
5	Positive psychology: part 1	• Review of the previous session—Recap of key concepts from Session 4, emphasizing the importance of managing cognitive errors and stress. • Importance of positive thinking and optimism—Discussion on how cultivating positive thinking and optimism can improve mental health, well-being, and resilience in daily life. • Training in positive thinking—Techniques for identifying and strengthening personal assets and developing a focus on positive traits to enhance self-esteem and resilience. • Q&A and group discussion—Facilitating participant reflections on applying positive thinking strategies and sharing experiences. • Group training activities—Interactive exercises to practice positive thinking, identifying personal strengths, and focusing on positive aspects of life.
6	Positive psychology: part 2	• Review of the previous session—Recap of key concepts from Session 5, focusing on the practice of positive thinking and its impact on well-being. • Techniques for replacing negative thoughts—Strategies for transforming irrational and negative thoughts into rational, positive alternatives to promote mental health. • Training in positive psychotherapy exercises—Practical application of techniques such as “Positive Reminiscence,” “Hope, Optimism, and Post-Traumatic Growth,” “Gratitude Writing,” and “Finding Meaning” to foster a positive mindset. • Q&A and group discussion—Participants discuss their experiences with the exercises, share insights, and address any challenges encountered. • Group training activities—Interactive exercises to practice the positive psychotherapy techniques and solidify the learning through real-life scenarios.

### Control condition

Mothers in the control group received standard care and did not participate in the resilience training. Upon completion of the follow-up assessments, they were provided with educational materials on resilience skills at the hospital.

### Measures

Data were collected using online questionnaires and forms. A socio-demographic and clinical assessment form, developed by the researchers, was utilized to gather information on the socio-demographic characteristics of the mothers of children with burn injuries (including age, occupation, educational level, and economic status) and the clinical-demographic details of their children (including age, gender, percentage of burns, degree of burns, cause of burns, hospitalization duration, and postoperative day). The outcome measures were as follows:

### Primary clinical outcome

Resilience was designated as the primary clinical outcome, based on the assumption that enhanced parental resilience may serve as a protective factor against child pain outcomes^36^. Resilience was assessed using the Connor-Davidson Resilience Scale (CD-RISC)^47^.

The CD-RISC consists of 25 items, each rated on a Likert scale from 0 (not true at all) to 4 (very true, nearly all the time). The total score can range from 0 to 100, with higher scores indicating greater resilience. The scale demonstrates strong internal consistency, with a Cronbach’s alpha of 0.89^47^. The scale has demonstrated good convergent validity. Factor analysis revealed five distinct factors within the scale^47^. The Persian version of the CD-RISC also exhibits high internal consistency (Cronbach’s alpha = 0.89) and sufficient validity^48,49^. In this study, the Connor-Davidson Resilience Scale showed high reliability with a Cronbach’s alpha of 0.82.

### Secondary clinical outcome

The researchers used the Visual Analog Scale (VAS) for pain assessment, which consists of a 10-centimeter-long vertical or horizontal line, with 0 indicating the absence of pain and 10 representing extreme or intolerable pain. The scale has demonstrated concurrent validity with the Numerical Pain Rating Scale (NPRS), ranging from 0.71 to 0.78, and test-retest reliability ranging from 0.71 to 0.99^50^. In Iran, the scale’s reliability has been reported to be 0.81^51^. In the present study, the reliability of the tool was assessed using Cronbach’s alpha, yielding a coefficient of 0.87.

### Statistical analysis

Data analysis was performed using SPSS software, version 22 (SPSS, Inc., Chicago, IL, USA). Descriptive statistics, including means and standard deviations for continuous variables and frequencies and percentages for categorical variables, were used to summarize the data. The normality of data distribution was first assessed using the Kolmogorov-Smirnov test to verify adherence to the normality assumption. To evaluate group homogeneity regarding demographic and clinical characteristics, the chi-square test (or Fisher’s exact test when appropriate) was applied for categorical variables, and the Mann-Whitney U test was used for continuous variables due to the non-normal distribution of the data. The Wilcoxon signed-rank test was utilized for assessing significant differences within groups, while the Mann-Whitney U test was employed to compare differences between groups. Missing data were addressed using the mean series imputation method in SPSS. For the purposes of data analysis and interpretation, the treatment groups were coded as intervention (0) and control (1).

In this study, a Generalized Estimating Equation (GEE) model was employed to assess the impact of the intervention on the improvement of children’s pain and mothers’ resilience. Given that the data on the effect of resilience training were collected over twelve consecutive measurements, the responses were both correlated and longitudinal. The GEE method is particularly suited for analyzing such correlated and longitudinal data. This method evaluated the effect of the intervention (resilience training) and calculated the variance matrix using the exchangeable correlation structure in the longitudinal context. The GEE technique is valuable for handling repeated measures or time-series data and for estimating causal models across panels or for entire datasets. It provides asymptotic estimates based on quasi-likelihoods, even when correlations between explanatory and dependent variables are unknown or when there are missing partial correlations^52^. Statistical significance was defined as a P-value of less than 0.05.

### Ethical considerations

This study was approved by the Ethics Committee of Shiraz University of Medical Sciences, with reference number IR.SUMS.REC.1398.1098. It was also registered in the Iranian Registry of Clinical Trials (Registration number: IRCT20201001048893N2, 8/12/2020, https://irct.behdasht.gov.ir/search/result?query=IRCT20201001048893N2). The study was conducted in adherence to the principles of the Declaration of Helsinki, and all participants provided written consent after being informed about the study procedures. The research methods were performed in accordance with the applicable guidelines and regulations. Prior to participation, eligible mothers of children with burn injuries were provided with detailed information about the study. Consent was obtained from both the mothers and, where applicable, the children. Participants were informed of their right to withdraw from the study at any time without facing any negative repercussions. To avoid any financial burden on the participants, their involvement in the study was at no cost. Confidentiality was strictly maintained; data were anonymized, and questionnaires were assigned numerical codes instead of using personal identifiers. Upon completion of the study, educational pamphlets on resilience skills were provided to the control group as a form of post-study support.

## Results

Of the 65 participants (children with burn injuries and their mothers) assessed for eligibility, 56 were found to meet the criteria. Six mothers had to be excluded from the study—three from each of the intervention and control groups—because their children were discharged before completing the training sessions. Consequently, 50 participants completed the study, with 25 in the control group and 25 in the intervention group.

The mean age of the mothers was 33.80 years (standard deviation [SD] = 8.47), while the mean age of their children was 9.64 years (SD = 3.29). Most mothers had a high school education (82%). 70% resided in rural areas, and 92% were unemployed (Table 2). Among the children, 52% were male, 54% had upper extremity burns, and 74% had second- or third-degree burns. Additionally, 44% of the children were admitted with burns covering more than 20% of their bodies. There were no significant differences in baseline sociodemographic and clinical characteristics between the intervention and control groups (*p* > 0.05).

**Table 2:** Demographic characteristics of samples (*N* = 50).

Group	Intervention (*n* = 25)	Control (*n* = 25)	Total (*n* = 50)	*P*-value
Variables				
Children’s age (year), mean ± SD	9.64 ± 3.29	9.32 ± 2.94	9.48 ± 3.092	0.718
Mother’s age (year), mean ± SD	34.76 ± 7.79	32.84 ± 9.15	33.80 ± 8.47	0.428
Gender, n (%)				0.396
Male	11 (44)	15 (60)	26 (52)	
Female	14 (56)	10 (40)	24 (48)	
Father’s Job, n (%)				0.122
Self-employed	11 (44)	13 (52)	24 (48)	
Employee	6 (24)	1 (4)	7 (14)	
Worker	8 (32)	11 (44)	19 (38)	
Mother’s Job, n (%)				0.609
Working	3 (12)	1 (4)	4 (8)	
Non-working	22 (88)	24 (96)	46 (92)	
Father’s education, n (%)				0.017*
Illiterate	1 (4)	8 (32)	9 (18)	
High school	14 (56)	12 (48)	26 (52)	
Diploma	3 (12)	4 (16)	7 (14)	
Academic degree	7 (28)	1 (4)	8 (16)	
Mother’s education, n (%)				0.182
High school	18 (72)	23 (92)	41 (82)	
Diploma	3 (12)	1 (4)	4 (8)	
Academic degree	4 (16)	1 (4)	5 (10)	
Economic status, n (%)				0.714
< 1 million Rials	8 (32)	12 (48)	20 (40)	
1–2 million Rials	10 (40)	8 (32)	18 (36)	
2–3 million Rials	4 (16)	3 (12)	7 (14)	
> 3 million Rials	3 (12)	2 (8)	5 (10)	
Place of living, n (%)				0.758
Urban	8 (32)	7 (28)	15 (30)	
Rural	17 (68)	18 (72)	35 (70)	

*P-value < 0.05.

The baseline characteristics of the participants are detailed in Tables 2 and 3. Most of the demographic and clinical characteristics did not show significant differences between the intervention and control groups, indicating a relative homogeneity among the study participants. However, a significant difference was observed in fathers’ education level (*p* = 0.017), which could be considered a potential influencing factor in interpreting the study findings.

**Table 3:** Clinical characteristics of samples (*N* = 50).

Group	Intervention (*n* = 25), n (%)	Control (*n* = 25), n (%)	Total (*n* = 50), n (%)	*P*-value
Variables				
Burning percentage				0.670
< 10	5 (20)	6 (24)	11 (22)	
10–20	10 (40)	7 (28)	17 (34)	
> 20	10 (40)	12 (48)	22 (44)	
Place of burning				0.890
Lower extremity	5 (20)	3 (12)	8 (16)	
Upper extremity	13 (52)	14 (56)	27 (54)	
Feet	2 (8)	2 (8)	4 (8)	
Hand	5 (20)	6 (24)	11 (22)	
Burn degree				0.567
2	3 (12)	1 (4)	4 (8)	
3	2 (8)	4 (16)	6 (12)	
4	1 (4)	2 (8)	3 (6)	
2 and 3	19 (76)	18 (72)	37 (74)	
Burning method				0.908
Hot drink	7 (28)	6 (24)	13 (26)	
Hot food	3 (12)	3 (12)	6 (12)	
Fire	4 (16)	4 (16)	8 (16)	
Petrol	4 (16)	2 (8)	6 (12)	
Acid	1 (4)	3 (12)	4 (8)	
Hot objects	2 (8)	1 (4)	3 (6)	
Electricity	2 (8)	2 (8)	4 (8)	
Firecracker	2 (8)	4 (16)	6 (12)	
Hospitalization (Days)				0.236
1	10 (40)	10 (40)	20 (40)	
2	6 (24)	11 (44)	17 (34)	
3	7 (28)	2 (8)	9 (18)	
4	2 (8)	2 (8)	4 (8)	
Surgery’s day				0.337
2	6 (24)	5 (20)	11 (22)	
3	4 (16)	5 (20)	9 (18)	
4	3 (12)	3 (12)	6 (12)	
5	3 (12)	2 (8)	5 (10)	
6	1 (4)	2 (8)	3 (6)	
7	0 (0)	1 (4)	1 (2)	
8	0 (0)	1 (4)	1 (2)	
No surgery	8 (32)	6 (24)	14 (28)	

The results of the GEE analysis revealed significant main effects of time on child pain intensity (B = − 0.84, *p* < 0.001) and maternal resilience (B = 3.99, *p* < 0.001), indicating that both child pain intensity and maternal resilience significantly improved over time. These findings suggest that, regardless of group membership, the passage of time had a substantial effect on both outcomes, with mothers showing increased resilience and children experiencing reduced pain intensity over the course of the study.

Moreover, the analysis revealed significant main effects of group on child pain intensity (B = 2.85, *p* < 0.001) and maternal resilience (B = − 3.05, *p* < 0.001). These results indicate that the intervention group demonstrated significantly greater improvements in both child pain intensity and maternal resilience compared to the control group. Specifically, when controlling for the effect of time, the intervention group exhibited a more pronounced reduction in child pain intensity and a greater increase in maternal resilience than the control group. These findings suggest that the intervention had a beneficial impact on both outcomes, beyond the natural changes that occurred over time.

**Table 4:** Estimated effects of group, time, and group × time interactions on dependent variables (Children’s pain and mothers’ resilience) using the generalized estimating equations (GEE) model.

Dependent variable	B	SE	Wald statistic	95%CI		*P*-value
				Lower	Upper	
Child pain						
Intercept	4.47	2.18	4.19	0.19	8.75	0.041*
Group	2.85	0.54	27.47	1.78	3.92	0.000***
Time	-0.84	0.09	78.76	-1.02	-0.65	0.000***
Group × Time	-0.05	0.13	0.15	-0.31	0.21	0.69^ns^
Mother resilience						
Intercept	29.44	7.58	15.09	14.59	44.29	0.000***
Group	-13.05	2.48	27.64	-17.91	-8.18	0.000***
Time	3.99	0.55	52.71	2.91	5.07	0.000***
Group × Time	-2.06	0.64	10.23	-3.33	-0.80	0.001***

B , model coefficient; SE, standard error; 95%CI , 95% confidence interval.

Parameter estimates are obtained from GEE models.

Treatment group: 0 = intervention, 1 = control.

The controlled variables in the GEE models included demographic and clinical characteristics such as children’s age, mother’s age, gender, parental education (mother’s and father’s), parental job (mother’s and father’s), burning percentage, burn degree, hospitalization duration, and surgery day.

ns = no significant; *P < 0.05, ***P ≤ 0.001.

The interaction between group and time for maternal resilience was statistically significant, indicating that the GEE analysis revealed a significant difference in maternal resilience between the intervention and control groups over time (B = − 2.06, *p* = 0.001). Specifically, this interaction suggests that the intervention group showed a greater improvement in maternal resilience over time compared to the control group. This finding highlights the effectiveness of the intervention in enhancing maternal resilience, with the benefits becoming more pronounced as time progressed.

**Fig. 2:**
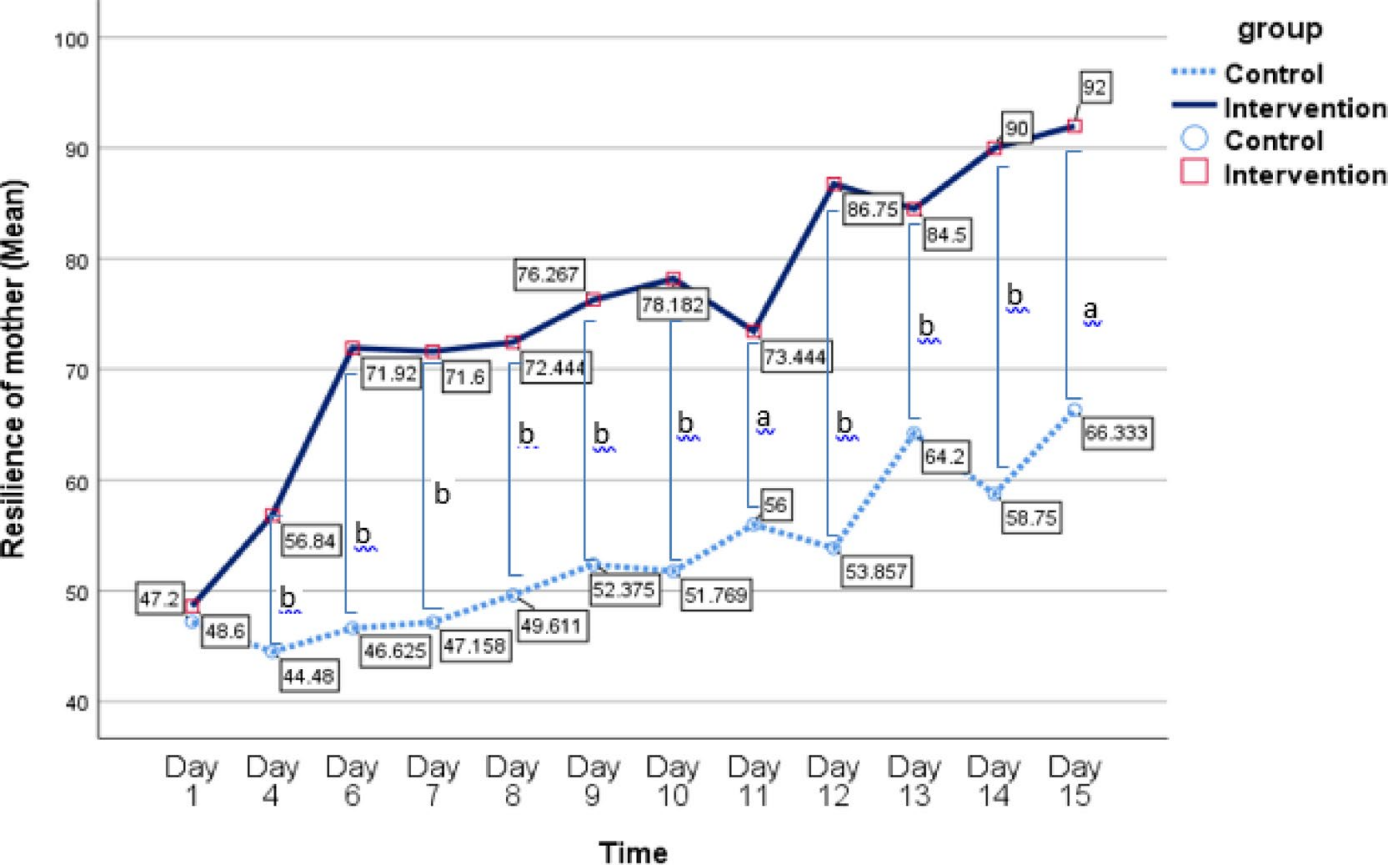
Mean changes in mother resilience across repeated measurement times for intervention and control groups. a: *P* < 0.05, b: *P* ≤ 0.001.

**Fig. 3:**
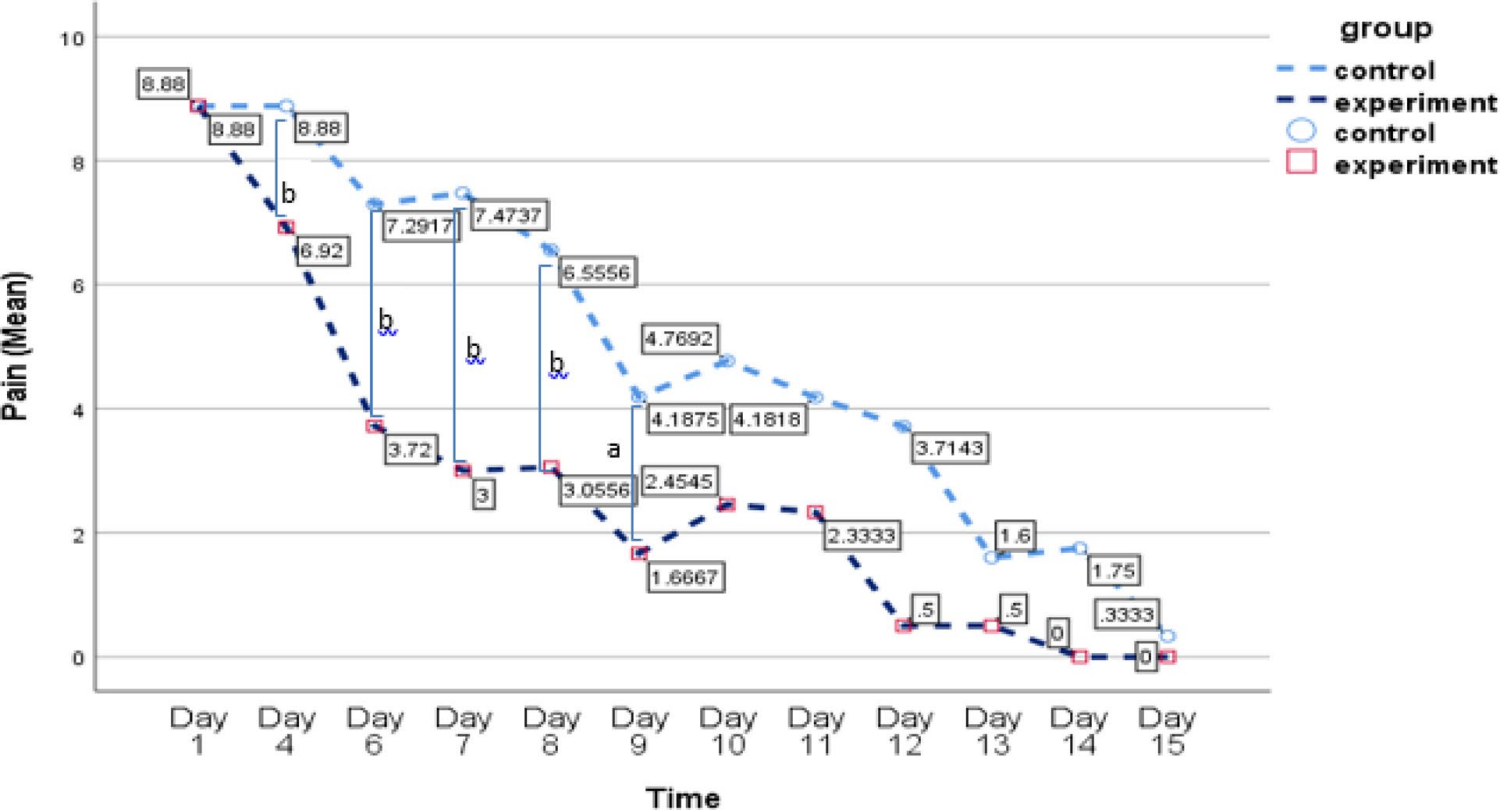
Mean changes in children’s pain across repeated measurement times for intervention and control groups. a: *P* < 0.05, b: *P* ≤ 0.001.

In contrast, the interaction between group and time for child pain intensity was not statistically significant (B = − 0.05, *p* = 0.69), suggesting that the improvement in child pain intensity over time did not differ significantly between the intervention and control groups. This lack of a significant interaction indicates that while pain intensity decreased for both groups over time, the intervention did not produce a significantly greater reduction in pain intensity compared to the control group. Additional details are provided in Table 4; Figs. 2 and 3.

Paired t-test analysis was conducted to compare participants’ scores between the first day’s assessment and subsequent assessments. For the control group, the analysis of mothers’ resilience scores revealed significant differences between the mean resilience score on the first day and the scores on the 11th day (t = − 4.01, df = 10, *p* = 0.002), the 13th day (t = − 5.29, df = 4, *p* = 0.003), and the 15th day (t = − 5.89, df = 10, *p* = 0.03). There was a significant reduction in pain intensity from the first day’s assessment to all subsequent pain assessments in children of both the control and experimental groups (*p* < 0.05). Additionally, a significant increase in resilience was observed in the experimental group between the first day’s assessment and all subsequent resilience assessments (*p* < 0.05).

While both groups showed improvements over time, the intervention group showed more pronounced and sustained improvements. This suggests that the resilience training program was an effective addition to the standard care regimen, emphasizing the importance of caregiver emotional support in pediatric pain management. These findings underscore the importance of integrating resilience-focused psychological support into routine burn care to optimize both maternal and child outcomes. They highlight the need for holistic, family-centered approaches in pediatric burn care settings to enhance coping mechanisms and overall recovery.

## Discussion

Given the high risk of psychological distress among mothers of children with burn injuries^31,32^ and its potential impact on their children’s pain^33,34^, it is crucial to assess effective psychological interventions. Therefore, the present study aimed to evaluate the effect of a resilience training intervention for mothers of hospitalized children with burn injuries on the resilience of mothers and the pain of their burnt children.

The results demonstrated that the intervention significantly improved maternal resilience, with sustained benefits observed throughout the study. Previous studies have shown that resilience training can enhance mental health in mothers facing various challenges, including intellectual disabilities^53^, leukemia^54^, and ADHD^55^. While studies directly investigating resilience training for mothers of children with burn injuries are limited, some research on psychosocial interventions for these parents suggests promising results. For example, Sveen et al. (2017) found that a six-week psychoeducational program reduced post-traumatic stress in parents of burned children^25^. Similarly, Shaygan et al. (2025) reported that resilience programs alleviated anxiety in mothers during the acute phase of hospitalization^56^, while Tully et al. (2022) associated improved resilience with reduced traumatic stress several months post-injury^57^. Furthermore, Hornsby et al. (2019) conducted a systematic review of psychosocial interventions, emphasizing the importance of early psychosocial support in promoting the psychological recovery of parents following their children’s burn injuries. According to this review, parent group counseling, a form of resilience training, has shown promise in enhancing maternal resilience^58^.

Dealing with a child’s burn injury is one of the most stressful experiences for parents^57^, especially mothers^14,59^, leading to elevated stress, anxiety, depression, and other emotional burdens^59^. These psychological reactions can negatively affect both the parents’ and children’s well-being. Previous research suggests that parental distress can increase the risk of low resilience in children^60^, while low resilience in pediatric burn survivors is linked to more intense symptoms in caregivers^61^. In this study, it seems that resilience training helped mothers to manage psychological stress, improve coping strategies, and enhance mental health support, benefiting both the mothers and their children. This study suggests that resilience training improved mothers’ psychological well-being and likely led to better outcomes for their children by alleviating parental distress, which in turn enhanced maternal resilience. While improvements in the child’s condition due to medical treatment may have contributed to the outcomes, resilience training seems to have had a positive effect on both mothers and children. Therefore, incorporating a family-based resilience approach in burn services is recommended to support both parents and children following trauma.

The resilience training group showed a significant reduction in child pain compared to its baseline. Furthermore, it exhibited a greater reduction compared to the control group, considering the main effect of the group. These findings suggest that enhancing maternal resilience may improve coping strategies and enhance children’s pain management. This highlights the broader benefits of resilience training beyond individual coping mechanisms. Previous studies indicate that parental behaviors significantly affect children’s pain during medical procedures^62–65^. For example, Brown et al. (2019) found that parental psychological distress impacts children’s pain during burn care, and acute psychological support for parents may reduce pain-related distress in children^30^. Additionally, Maia Brasil et al. (2012) emphasized the role of family resilience and emotional support in reducing children’s pain post-burn injury^66^. Psychoeducation for parents not only reduces their stress but also strengthens family cohesion and aids the child’s recovery^67^. Although the impact of resilience training on children’s pain had not been fully explored, this study addresses this gap by demonstrating its positive effects.

In the control group, where mothers did not receive resilience training, a reduction in children’s pain was observed, though less pronounced than in the intervention group. Both groups received standard care, including pharmacological treatments, which are critical in managing pediatric burn pain^68,69^. However, pain reduction in the control group could also be attributed to the natural healing process^70^, tissue regeneration^70^, and parental presence during procedures^71^. Repeated exposure to painful procedures, such as dressing changes, can reduce both emotional and physiological pain responses over time^72^. While these factors contributed to pain relief in the control group, resilience training for mothers led to more significant improvements in pain management. Effective pain management is essential, as untreated pain can delay wound healing^73,74^ and lead to chronic pain or post-traumatic stress^75,76^. Pharmacological interventions remain the cornerstone of effective pediatric burn pain management^68,69^. However, pharmacological treatments can have limitations, including insufficient pain control, respiratory depression, and excessive sedation^77^. Additionally, children often respond less effectively to medical treatments compared to adults, highlighting the importance of complementary or alternative therapies^78^. Non-pharmacological pain management strategies, such as resilience training for mothers, offer an effective, cost-efficient, and practical approach, reducing reliance on painkillers while enhancing children’s pain relief.

Our study supports Brown et al. (2019), showing that enhancing psychological support for parents of burn-injured children alleviates procedural pain-related distress in these children. Resilience training for mothers in the experimental group reduced their psychological distress, which in turn decreased their children’s pain. While the control group also showed pain reduction, adding resilience training to standard care was more effective. This effect may be due to improved parental coping, which creates a more supportive environment that reduces anxiety and enhances pain management. Resilient parents can also model adaptive coping behaviors, influencing their children’s pain perception and tolerance. This study highlights the importance of integrating resilience training for parents in pediatric burn care.

A key limitation of this study was the high attrition rate due to child discharges, which may have influenced the results. Additionally, cultural and economic factors, which could affect coping mechanisms, were not considered and may have impacted the outcomes of resilience training and pain management. Future research should address these variables to better understand their influence. The use of mean imputation for missing data may have underestimated variability, so more robust methods like multiple imputation or maximum likelihood estimation are recommended. The sample was predominantly from rural areas with limited resources, and many children had upper extremity burn injuries, limiting generalizability. The small sample size also affects the robustness of the conclusions. Future studies should include larger, more diverse populations. Furthermore, the study did not isolate the effects of pharmacological treatments, which should be analyzed in future research to better evaluate their role in pain reduction. Finally, exploring other psychosocial interventions and their impact on maternal resilience and child outcomes would be valuable.

## Conclusion

Parental psychological distress following a child’s burn injury can impact both the child’s physical recovery and the ongoing psychological well-being of both the child and the mother^79^. Therefore, implementing effective psychological interventions for mothers is crucial. In the present study, resilience training for mothers led to a significantly greater improvement in maternal resilience over time compared to the control group. However, although pain intensity decreased significantly in both groups, the reduction in the intervention group was greater but not statistically significant compared to the control group over time. The training likely improves maternal coping strategies and mental health support, which fosters a more supportive environment for the child. This, in turn, may reduce the child’s anxiety and pain perception.

The study supports integrating resilience training into family-centered pediatric burn care, offering mothers essential psychological support to manage distress and adapt to their child’s condition. This approach is accessible, cost-effective, and suitable for rehabilitation. However, further research is needed to better address the psychosocial needs of these families, with a focus on tailoring interventions based on individual circumstances and burn injury characteristics.

## Data Availability

Upon a reasonable request from scientists, the associated data can be made available by reaching out to the corresponding author via email. Moreover, researchers interested in obtaining the intervention manual may contact the corresponding author for further information.
